# *Announcement:* National Child Passenger Safety Week — September 17–23, 2017

**DOI:** 10.15585/mmwr.mm6636a8

**Published:** 2017-09-15

In 2017, National Child Passenger Safety Week is being observed during September 17–23. In the United States, motor vehicle–related injuries are a leading cause of death among children (*1*). In 2015, a total of 663 passenger-vehicle occupants aged ≤12 years died as a result of a crash (*2*), and nearly 132,000 were injured (*1*). Among the children who died in 2015, 35% were known to be unrestrained (*2*). To keep child passengers as safe as possible, drivers should use age- and size–appropriate restraints for all child passengers until adult seat belts fit properly (lap belts should lay across upper thighs, not abdomen, and shoulder belts should lay across the middle of the shoulder and chest, not the neck or face) and follow the American Academy of Pediatrics’ child passenger safety recommendations (*3*). Children aged <13 years should be properly restrained in the back seat.

As part of National Child Passenger Safety Week, September 23 has been designated “National Seat Check Saturday.” On this day, drivers with children who ride in car seats or booster seats are encouraged to visit a child safety seat inspection station to have a certified technician inspect their car seat for proper installation and proper use, free of charge. Additional information and an inspection station locator are available from CDC at https://www.cdc.gov/motorvehiclesafety/child_passenger_safety and the National Highway Traffic Safety Administration at https://www.safercar.gov/parents/index.htm. Campaign promotional materials in English and Spanish are available at https://www.trafficsafetymarketing.gov/get-materials/child-car-safety/child-passenger-safety-week.
