# Erratum: Vol. 66, No. 36

**DOI:** 10.15585/mmwr.mm6639a5

**Published:** 2017-10-06

The announcement “Childhood Cancer Awareness Month — September 2017,” on page 963, should have included the following as the third and last reference:

**Oeffinger KC, Mertens AC, Sklar CA, et al.; Childhood Cancer Survivor Study. Chronic health conditions in adult survivors of childhood cancer. N Engl J Med 2006;355:1572–82.**

